# A modified generative adversarial networks with Yolov5 for automated forest health diagnosis from aerial imagery and Tabu search algorithm

**DOI:** 10.1038/s41598-024-54399-w

**Published:** 2024-02-27

**Authors:** Prabhu Jayagopal, Kumar Purushothaman Janaki, Prakash Mohan, Upendra Babu Kondapaneni, Jayalakshmi Periyasamy, Sandeep Kumar Mathivanan, Gemmachis Teshite Dalu

**Affiliations:** 1 School of Computer Science Engineering and Information Systems, Vellore Institute of Technology, Vellore, 632014 India; 2 School of Computer Science and Engineering, Vellore Institute of Technology, Vellore, 632014 India; 3 School of Computing, Department of Computer Science and Engineering, Bharath Institute of Higher Education and Research, Bharath Institute of Science and Technology, 173, Agaram Main Road, Selaiyur, Tambaram, Chennai, 600073 Tamil Nadu India; 4 School of Computer Science and Engineering, Galgotias University, Greater Noida, 203201 India; 5 Department of Software Engineering, College of Computing and Informatics, Haramaya University, POB 138, Dire Dawa, Ethiopia

**Keywords:** Forest fires, Modified generative adversarial networks, YOLOv5, Tabu search algorithm, Deep learning, Aerial imagery, Cancer, Diseases, Health care, Medical research

## Abstract

Our environment has been significantly impacted by climate change. According to previous research, insect catastrophes induced by global climate change killed many trees, inevitably contributing to forest fires. The condition of the forest is an essential indicator of forest fires. Analysis of aerial images of a forest can detect deceased and living trees at an early stage. Automated forest health diagnostics are crucial for monitoring and preserving forest ecosystem health. Combining Modified Generative Adversarial Networks (MGANs) and YOLOv5 (You Only Look Once version 5) is presented in this paper as a novel method for assessing forest health using aerial images. We also employ the Tabu Search Algorithm (TSA) to enhance the process of identifying and categorizing unhealthy forest areas. The proposed model provides synthetic data to supplement the limited labeled dataset, thereby resolving the frequent issue of data scarcity in forest health diagnosis tasks. This improvement enhances the model's ability to generalize to previously unobserved data, thereby increasing the overall precision and robustness of the forest health evaluation. In addition, YOLOv5 integration enables real-time object identification, enabling the model to recognize and pinpoint numerous tree species and potential health issues with exceptional speed and accuracy. The efficient architecture of YOLOv5 enables it to be deployed on devices with limited resources, enabling forest-monitoring applications on-site. We use the TSA to enhance the identification of unhealthy forest areas. The TSA method effectively investigates the search space, ensuring the model converges to a near-optimal solution, improving disease detection precision and decreasing false positives. We evaluated our MGAN-YOLOv5 method using a large dataset of aerial images of diverse forest habitats. The experimental results demonstrated impressive performance in diagnosing forest health automatically, achieving a detection precision of 98.66%, recall of 99.99%, F1 score of 97.77%, accuracy of 99.99%, response time of 3.543 ms and computational time of 5.987 ms. Significantly, our method outperforms all the compared target detection methods showcasing a minimum improvement of 2% in mAP.

## Introduction

Deforestation and species extinction have been linked to global climate change, according to growing data over the past ten years. In addition to triggering severe weather events like floods, droughts, and storms, temperature changes have a definite effect on ecosystems. The ability of animals to adjust to changes may be compromised in such a setting^1^. Additionally, due to extreme heat and storms, 110,000 hectares of forest died in 2018, equivalent to 150,000 football fields or one-fifth of Wales. Several species that formerly lived in active forests have been forced to relocate because of deforestation and dying trees. On the other hand, parasitic wasps, fungi, bacteria, and predatory beetles all grow on decaying wood. Pests like bark beetles and cardinal beetles can make a home in dead tree trunks before attacking healthy trees^2^.

Furthermore, fire tragedies happen more frequently because dead wood is dry and combustible^3,4^. The incorrect treatment of dead wood in forests is a major cause of California's wildfire issue. Both were expensive because the fire soon spread to surrounding villages and killed nearby healthy trees. Forest fires also cause significant pollution; for instance, wildfire smoke in Indonesia is thought to have contributed to 100,000 premature deaths in 2015^5^. Technology for monitoring forest regions has improved quickly in recent decades because of the above implications on ecosystems, people, and homes. For instance, the cost of forest monitoring has decreased to remote aerial sensors or satellite images^6,7^, enabling monitoring without human intervention. This technique can be used by experts in forests and disaster management to both monitor forests in real time and prepare for natural disasters^8^.

On the other hand, experts' attention has recently been drawn to the insect issue, one of the natural disasters linked to climate change^9,10^. To stop pine wood nematode (PWN) from posing a threat to forests, the Canopy Health Monitoring (CanHeMon) project conducted earlier research to identify dead trees in forests and stop them from acting as PWN vectors. A Tabu Search approach may accurately identify specific tree tops that are degrading when used to remote sensing data^11^. The algorithm searches a set of restricting variables and a set of occurrences to find the distribution with the highest entropy. The method can remove irrelevant factors, leaving just those highly connected with dead trees and high levels of disorder, as higher entropy indicates. Nonetheless, the results showed that a smaller sample size increased the rate of false positives. When smaller sample sizes were considered, the rate of properly identifying positive cases fell from 80 to 65%. This could indicate that there needed to be more examples of little trees in the training set or that the algorithm needed to be built to recognize small objects.

GANs are an effective method for creating synthetic data and enhancing the functionality of various computer vision applications. We adapt the traditional GAN design in this work to meet the specific requirements of diagnosing forest health. The upgraded GANs are trained on a large dataset of aerial images from various forest types, seasons, and environmental conditions. Furthermore, the YOLOv5 object identification framework identifies and localizes potential forest health issues such as diseased trees, insect infestations, and other anomalies directly from aerial data. Because of its real-time performance and greater accuracy, the YOLOv5 algorithm is well-suited for large-scale forest monitoring applications.

This study addresses many fundamental issues inherent in traditional techniques of assessing forest health. We plan to generate synthetic yet realistic forest images using a Modified GAN that spans health states and environmental variables. In conjunction with real-world aerial images, this synthetic dataset provides a thorough training source for our YOLOv5-based object detection algorithm. The real-time detection capabilities of YOLOv5 enable the exact identification and localization of various forest health indicators, such as diseased trees, pest infestations, and environmental stresses.

To summarize, it is critical to strike a compromise between detection accuracy and calculation speed when updating the model. A good detection approach should strive to consider the two points mentioned above. YOLOv5, which is based on YOLOv4 and includes four versions: s, m, l, and x, is the most prevalent YOLO series detection method. YOLOv5x is a huge and computationally intensive program. Although YOLOv5s and YOLOv5m are faster, they are not as accurate. In terms of total parameters and total floating-point operations per second (FLOPS), YOLOv5l outperforms YOLOv4 in terms of speed and precision. For the reasons stated above, we adjusted YOLOv5l to reflect the features of UAV aerial photos in order to improve the model's detection performance.

This paper's main contribution are as follows:

- Enhanced Object identification Accuracy: By integrating YOLOv5 into the MGAN framework, aerial imagery's object identification accuracy is improved. This is important for recognizing and evaluating a range of forest health indicators, including illnesses, deforestation, and fire damage.
- Optimization of Limited Labeled Data: MGANs produce synthetic data, which helps alleviate the lack of labeled training samples. This is especially helpful for diagnosing forest health issues, where labeled data may be hard to come by or prohibitively expensive.
- Furthermore, YOLOv5 integration provides real-time object identification, allowing the model to recognize and pinpoint a wide range of tree species and potential health issues with extraordinary speed and precision. Due to its effective architecture, YOLOv5 may be installed on low-resource devices, enabling on-site forest monitoring applications.
- Utilize the TSA to help us identify unhealthy forest regions. The TSA technique effectively examines the search space, ensuring the model converges to a near-optimal solution, improving disease diagnosis precision and lowering false positives. Using a huge dataset of aerial images of various forest environments, we tested our MGAN-YOLOv5 technique.

The remaining sections of this article is organized as follows: An overview of related initiatives in automated forest health diagnosis and forestry computer vision applications is provided in Section "Literature survey". Section "Proposed system" details the tools and processes, including dataset gathering, model construction, and evaluation measures. The results of the experiment are covered in Section "Result and discussion". Section "Conclusion" concludes by summarizing the contributions and outlining the goals for further research in this fascinating field.

## Literature survey

An improved Mask R-CNN Deep Learning technique was developed by Sani-Mohammed et al.^12^ to recognize and separate standing dead trees in a mixed dense forest. This was accomplished by utilizing 195 images from a short training dataset using CIR aerial images. To get beyond the limitations of training datasets, transfer learning, and the image augmentation technique are initially examined. Then, considering the architecture of our model and the special collection of data we were dealing with (dead trees in images), we carefully chose hyperparameters. The performance of our model was then carefully scrutinized using a different test dataset that wasn't used in the deep neural network's training to confirm its applicability.

According to a study by Martin et al.^13^, the decomposition of trees and the storage of carbon within ecosystems and their cycles are society's most critical issues, including well-known organizations like the United Nations (UN). Large-scale forest diebacks are a potential worst-case scenario that could exacerbate climate conditions, and the degradation of trees and their impact on carbon storage and sequestration are particularly crucial. Therefore, for efficient forest management, encouraging a healthy lifestyle, and informing ecological decisions and policies, having a good grasp of the state and diversity of trees within a forest is essential. This information can be particularly useful for precisely estimating the carbon content of forests and timber and identifying and counting the number of dead trees in a forest to assess its general health.

A study was done by Zhang et al.^14^ to map the dead trees brought on by pinewood nematode outbreaks. The RF algorithm stood out among the other three when they compared them. A Geographic Information System (GIS) database, Sentinel-2 images with a 20-m resolution, and a digital elevation model created from radar data with a 30-m solution were the three datasets used in the study. This study was carried out in the Chinese province of Hubei's Dangyang City. Using a retrained Mask RCNN technique and a transfer learning strategy, Chiang et al.^15^ established a new framework for automatically recognizing dead trees from aerial images. In this work, we applied our approach to large datasets of aerial images and examined eight improved models.

Natesan et al.^16^ innovative UAV-based tree species categorization method utilizes the remaining neural networks. The artificial neural network was trained using the UAV images obtained over three years. The categorization accuracy for two distinct tree species was 80% and 51% in two testing sets, respectively. Modica et al.^17^ semi-automatic method with classification F-scores for olive and bergamot ranging from 0.85 to 0.91 was recommended for processing multispectral UAV images to identify and extract olive and bergamot tree tops to create vigor maps for precision agricultural applications.

Barmpoutis et al.^18^ thoroughly examined the algorithms used by several optical remote sensing systems to detect flame and smoke. Additionally, they provided an overview of the technology used in terrestrial, airborne, and spaceborne early fire warning systems. The research examined various models to obtain accurate fire detection under challenging circumstances. The study focused on the advantages and disadvantages of optical remote sensing-based fire detection systems to promote further investigation into the development of early warning fire systems.

Qingyun et al.^19^ introduced a cross-modality fusion transformer (CFT) and an attention mechanism for efficient cross-modal feature fusion. This approach uses the transformer architecture to extract visual features, which frees up the network to focus on a wide range of contextual data. Furthermore, an attention mechanism encourages the simultaneous merging of intra- and intermodal information. As a result, multispectral targets are more easily detected in aerial images. Research has proven this method's dependability and adaptability to various datasets.

In aerial images, targets have been seen to congregate in groups, according to Yang et al.^20^. They developed the clustered detection (ClusDet) network to address this problem. The cluster proposal subnetwork (CPNet), the scale estimation subnetwork (ScaleNet), and the specialized detection network (DetecNet) are the three primary parts of this network. During initial surveillance, the network prefers recognizing aggregated regions over single targets. By segmenting and feeding minute targets to the sophisticated detector for later identification, this method addresses the difficulty of consolidating small targets and rectifying uneven distribution in UAV images.

A feature fusion and scaling-based single shot detector (FS-SSD) was proposed by Liang et al.^21^ as a quick and precise method for detecting small objects from aerial perspectives. Average pooling was added to the feature fusion module as part of the SSD detector's improvement, and a new branch was added to the deconvolution module. A particular feature pyramid was created as a result of all these changes. Further, increasing detection accuracy is the method's incorporation of the spatial relationships between items and the detection task.

To advance UAV multitarget detection tasks, Jiao et al.^22^ proposed advanced target detection methods have been instrumental. These algorithms have significant memory and processing needs, making implementation in low-power image processors, such as edge devices, challenging. This issue has been resolved with the introduction of YOLO series detection networks. This series's current number of models is eight official versions and numerous branch variations.

### Limitations for existing system Limited resolution:

A significant limitation of the existing system is that it is very dependent on the representativeness of the training data, which may make it difficult for it to generalize to unexpected environmental circumstances or novel manifestations of forest health issues. Accuracy of the system also depends on the quantity and quality of high-resolution aerial imagery, which presents problems in areas with a dearth or poor quality of such data. In some situations, the accuracy of automated diagnoses is hampered by YOLOv3's intrinsic constraints, especially when it comes to managing small or densely packed forest health indicators. The integrated system's adoption in resource-constrained contexts may be limited by its computing resource requirements. Ultimately, the complexity of the system may still make it difficult to comprehend how the model makes decisions, even with efforts to improve interpretability. In order to improve the system and create automated forest health detection apps that are more dependable and strong, these constraints must be addressed.

### Problem identification

The existing aerial image technique for assessing forest health may require assistance in properly and precisely categorizing various forest health categories. Because the system's algorithms are insufficient to manage intricate fluctuations in the vegetation and surroundings, incorrect classification and assessments may occur. The current method may require assistance when utilized in larger forest areas or when confronted with a huge amount of aerial imagery. Due to the inability to scale, it may be difficult to efficiently examine broad geographic areas or manage a significant volume of data, making diagnosis time-consuming. The existing system's training data may require more diversity, limiting the model's generalizability. It is less useful for assessing forest health in novel and diverse scenarios since it does not adequately represent different forest types, health concerns, and geographic locations. Early detection of forest health issues is crucial for implementing timely treatments and halting the spread of diseases or pests. The existing mechanism may be unable to accomplish this. It may concentrate recognizing severe or noticeable symptoms while neglecting less obvious but crucial signs of diminishing forest health.

## Proposed system

**Figure 1:**
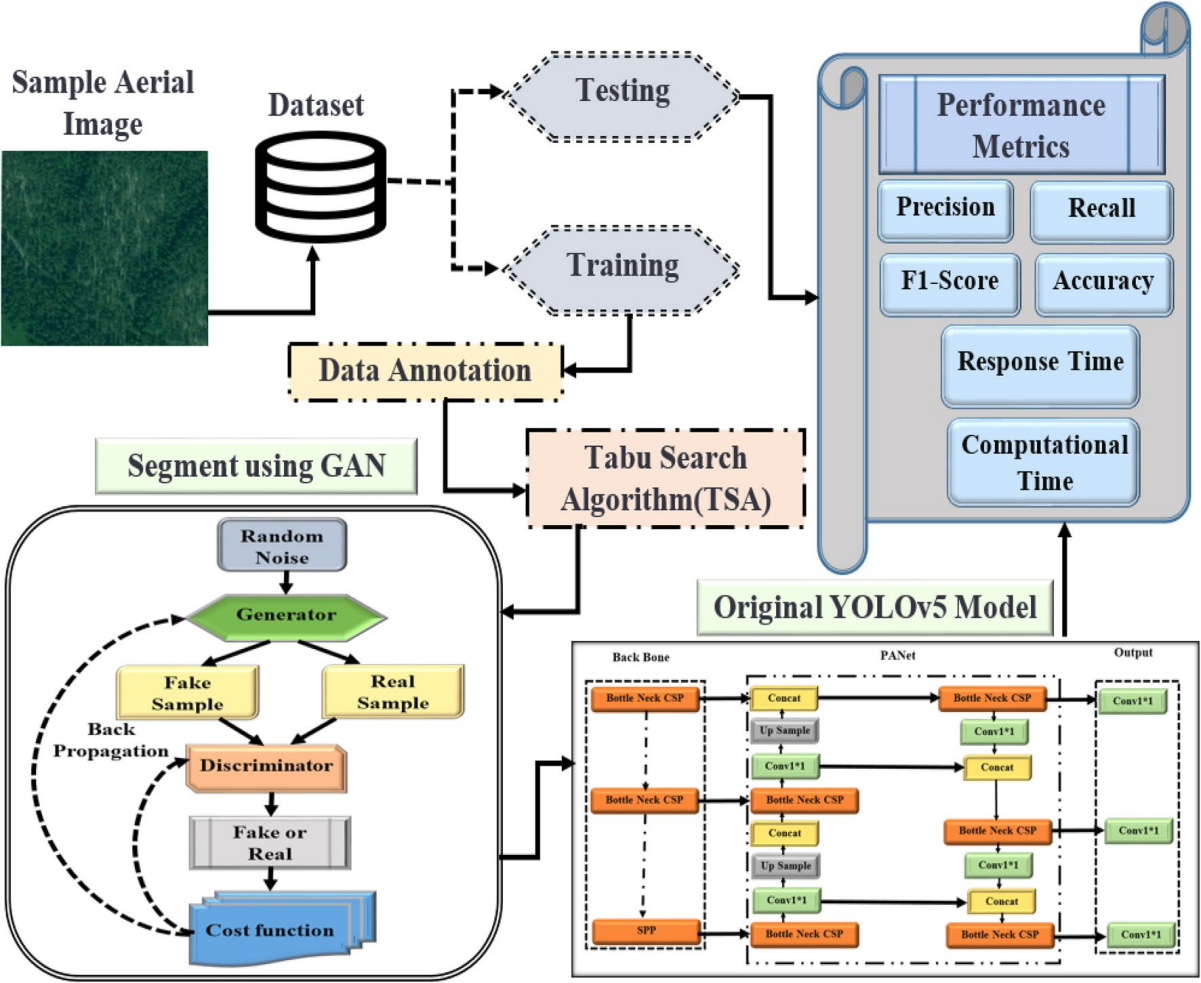
Proposed method of MGAN-YOLOV5.

In this section, we go into great depth about how we acquired the materials for our trials and modified the MGAN-YOLOV5 algorithm to produce promising results. The research area is also briefly summarized. The block diagram for the MGAN-YOLOV5 approach is shown in Fig. 1.

### Dataset description

**Figure 2:**
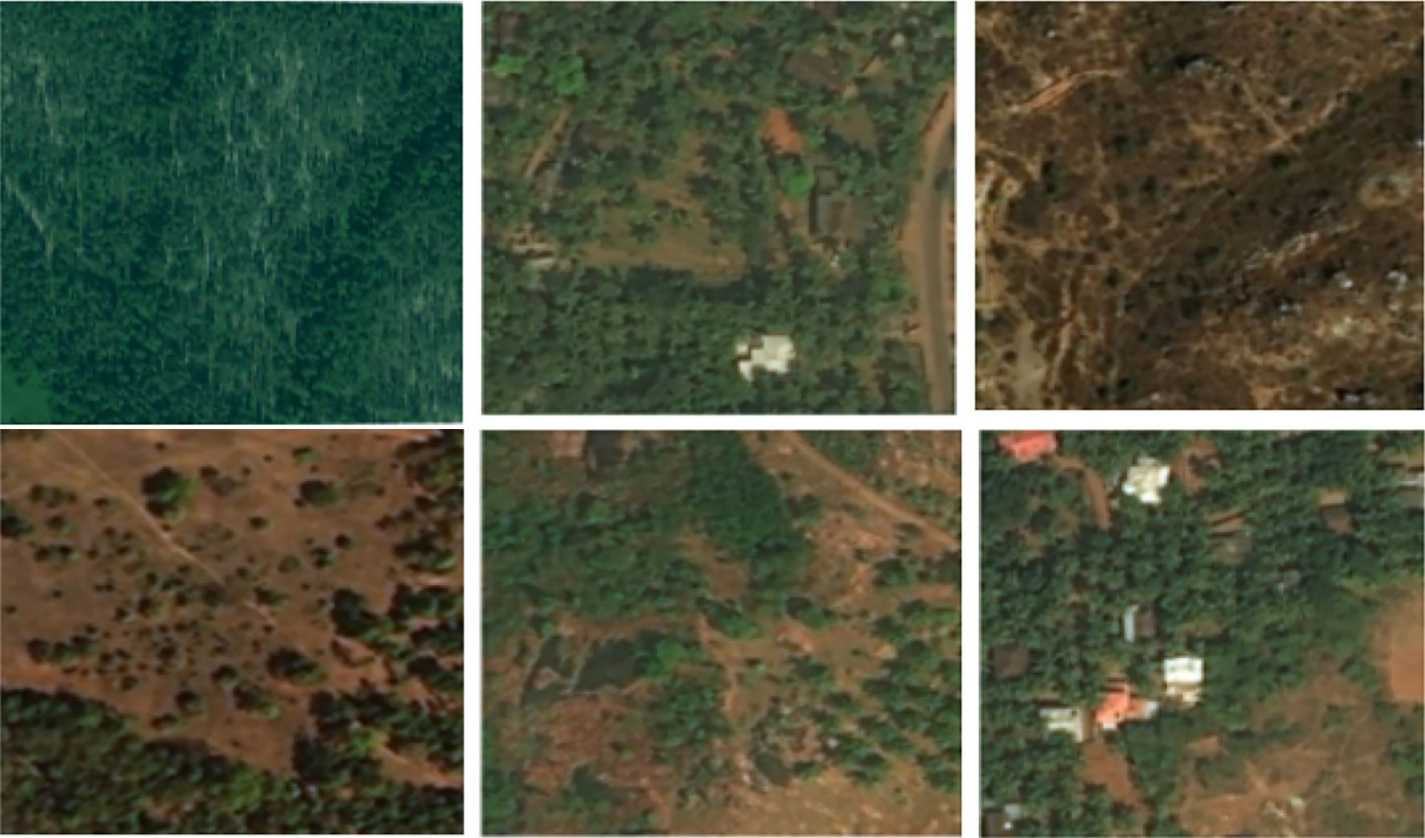
Dataset images.

On May 15, 2019, aerial images were used to acquire the data, which was then recorded in a 10 GB merged tiff file from Scotland's Wood of Cree. The file was too large for most of our tools, so we used the Gridsplitter plugin for QGIS to divide it into 40 × 10 patches, with an average size of 800 by 800 pixels each. Over 300 images of dead trees were chosen from the original data and used as the foreground objects in our synthetic dataset. Then, after randomly rotating and adjusting the luminance of these images, we positioned them on 63 suitable backgrounds that were likewise derived from the raw data. A mask was produced by painting the background black and adding different colors to the dead trees. The target items' x and y coordinates were gathered and stored in a COCO format annotation file. After adding 5000 patches to the original dataset of 225 patches, 5000 annotations were created in hours. Figure 2 shows the images from the dataset.

### Dataset annotation

The network architecture would need to learn, train, and test the predictions utilizing ground truth patterns to construct DL models. As a result, we made a great effort to accurately record every dead tree crown we came across on each patch of the images. We examined trees more significant than 50% of their total size to annotate the boundaries of the image patches. The image patches' estimated tree sizes range from 7 by 7 to 18 by 18 pixels. The VGG Image Annotator (VIA), an open-source web-based image annotator that does not require additional libraries or installs, was used to annotate each image in the three folders. Contemporary web browsers like Chrome and Firefox support the offline use of this software. We exported our annotated dead tree tops as a JSON file to facilitate our processes. A minimum of one dead tree and 70 annotations are present in each annotated image shown in Table 1^23^.

**Table 1:** Details of the labeled dataset.

Description	Images
File size	10 GB (tiff file)
Splitted patches	40 × 10
Average pixels size	800 × 800
No of dead trees	300
Suitable background	63
Extended patches	5000

### Tabu search algorithm (TSA)

Over the last two decades, TS algorithms have been used in a variety of actual combinatorial optimization applications. Optimality's challenge can be solved in a variety of ways, including minimum cost, minimum distance, maximum throughput, and so on. There are various applications relating to scheduling, telecommunications, and character reorganization in natural processing languages. In such cases, tabu search provides an optimal or near optimal solution. The tabu searches, invented by Fred Glover, are used to a wide range of situations. TS can be used directly in a variety of decision situations without the need for any mathematical transformations^24^. Artificial intelligence is one of the domains that the TS algorithm uses as it explores various parts of the search field. Therefore, applying intelligence improves the TS's problem-solving abilities and reactivity to memory-related problems like scheduling and job shop problems. It approached the problem at hand directly and iteratively.

The achievement of adaptive motion makes it possible to avoid local optima using the metaheuristic method known as TS Evaluating earlier solutions is temporarily postponed as TS deliberately selects new search directions. The following list includes each of the Tabu Search's essential elements^25^.

**Algorithm 1:**
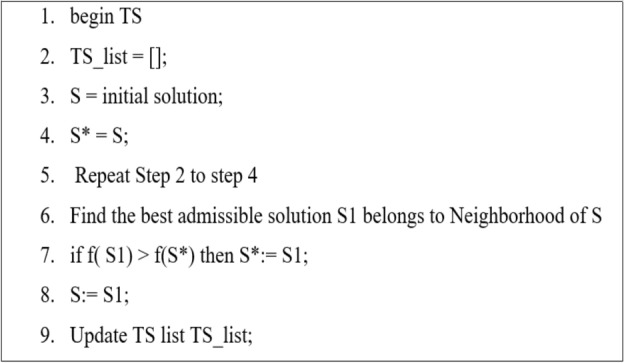
Tabu Search Algorithm.

Tabu List: This instrument provides the algorithm with short-to-medium-sized memory. The List "remembers" past searches and blocks moves. Tabu Moves are the name given to these disabled movements.

Tenancy Period: The duration (number of iterations) after which the Tabu List disables the Tabu Moves.

**Figure 3:**
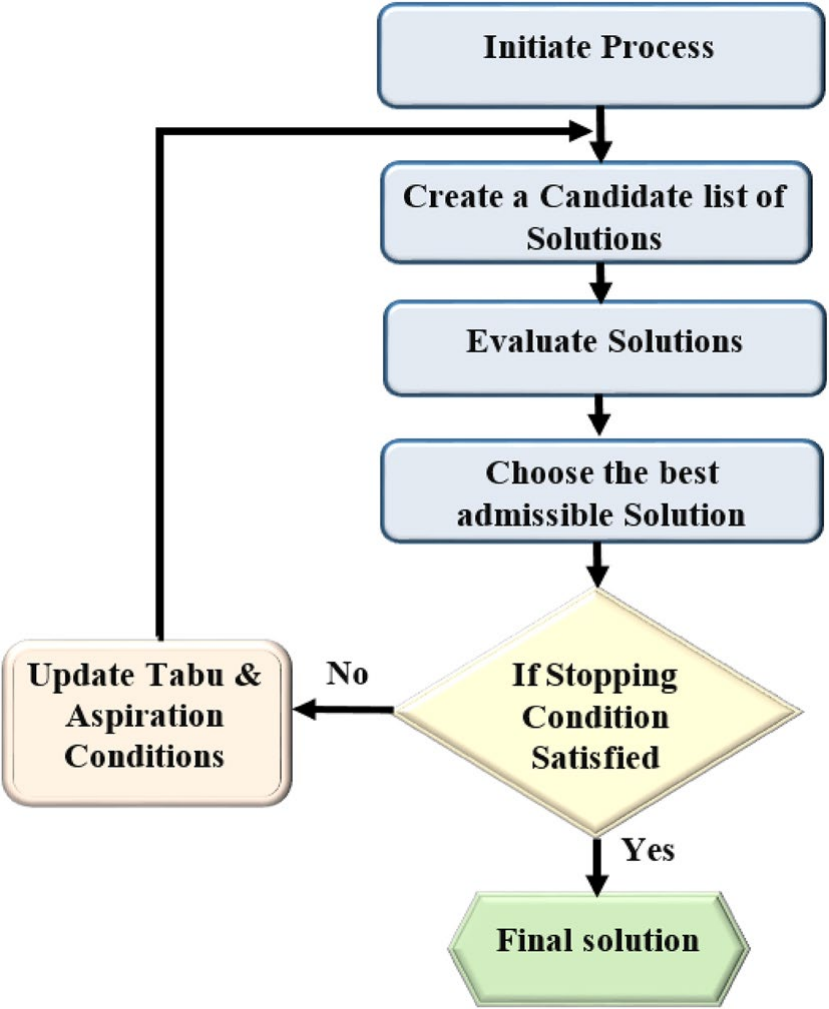
Flow chart of TSA.

Taha provides a detailed and general technique for the TS Algorithm as shown in Fig. 3.

- Step 0: Choose a starting solution ${m}_{0}\in M$. Choose a list tabu size and initialize the Tabu List with ${B}_{0}=\mathrm{\varnothing }$. Decide on k = 0.
- Step 1: Find the neighborhood feasibility $A\left({m}_{k}\right)$ that excludes the tabu list ${B}_{k}$ inferior members.
- Step 2: Choose the next movement ${m}_{k+1}$ from $A\left({m}_{k}\right)$ or ${B}_{k}$ if a better option exists, and update ${B}_{k+1}$.
- Step 3: If a termination condition is met, stop; otherwise, $k=k+1$ and return to 1.

### Image segmentation using GAN

**Figure 4:**
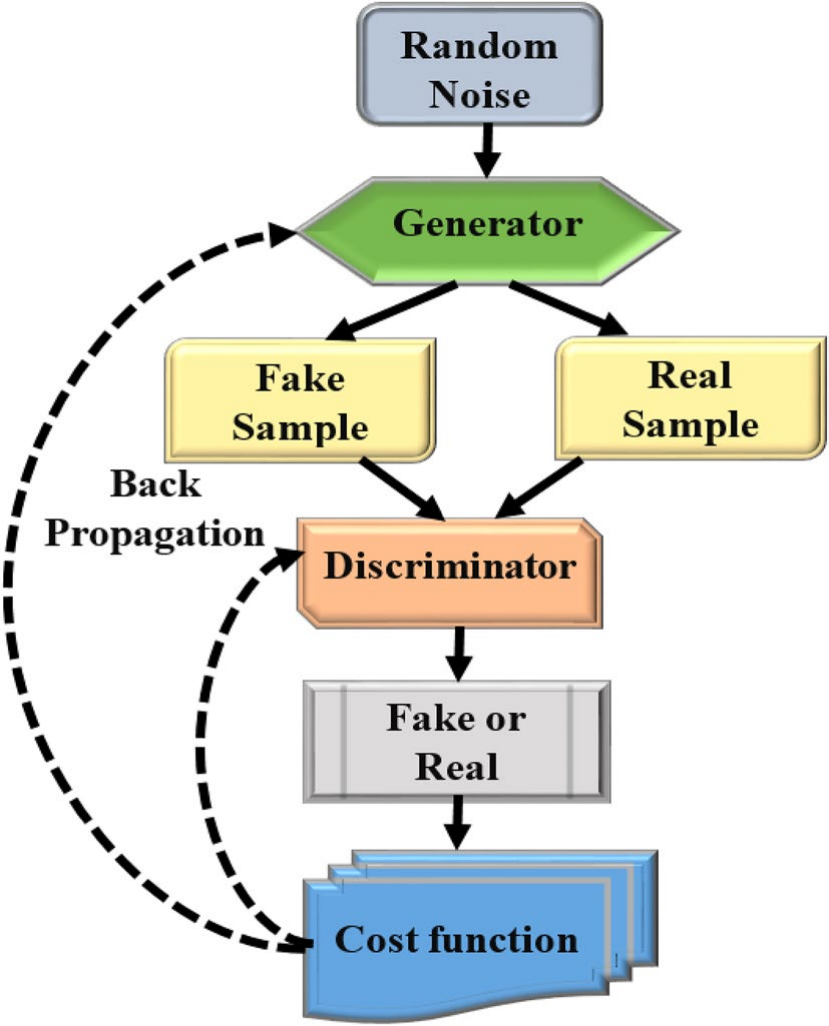
Architecture of modified generative adversarial network (MGAN).

Network of adversarial producers GAN has recently attracted a lot of attention since it can generate new samples by learning the probability distributions of the input data comparable to the training examples. Unlike other deep learning models, the GAN consists of only two networks: the generating and the discriminative as depicted in Fig. 4. As suggested by its name, GAN uses random noise to create new samples that resemble the original ones. It can also learn probability distributions from a dataset. The two networks compete against one another during the GAN training process to function as efficiently as possible. But, to "trickle" the discriminate network. The generative network must provide increasingly realistic samples; conversely, discriminate networks must learn how to recognize bogus images produced by the generative network. We can create new instances using the generating network for the trained GAN and extract or classify features using the discriminate network. The GAN's loss function is described as follows:

1 $$\mathop {\min }\limits_{K} \,\mathop {\max }\limits_{M} V\left( {M,K} \right) = F_{{y \sim p\,data^{\left( y \right)} }} \left[ {\log M\left( y \right)} \right] + F_{{z \sim p_{z}^{\left( z \right)} }} \left[ {\log \left( {1 - M\left( {K\left( z \right)} \right)} \right)} \right]$$

where p data represents the distribution of the real data (often real images), pz represents the distribution of the input noise, M represents the discriminating network, K represents the generative network, Y represents the input from the actual data, and z represents the input from the random noise. The discriminate network must recognize bogus samples to achieve $M\left(y\right)\to 1$ and $M\left(K\left(Z\right)\right)\to 0$, whereas the generative network must create realistic examples to get $M\left(K\left(Z\right)\right)\to 1$.

The original GAN has undergone several recent changes. The Deep Wasserstein GAN, which uses the Wasserstein distance to address the problem of vanishing gradients in the loss function, and the CycleGAN are a few examples of generative adversarial networks. These versions highlight the many GAN technological breakthroughs^26^.

In DCGAN, CNN replaces the multilayer perceptron previously utilized in GAN. Our method uses fractional-stride convolutions of the generative network to transform the low-dimensional input vector into a high-dimensional image. The discriminate network additionally uses stride convolutions to reduce the size of the input image and transform it into a range of (0,1), which stands for the likelihood that the input is accurate data. Random noise, commonly called z, is fed into the CGAN. The CGAN loss function is defined as follows when given y:

2 $$\mathop {\min }\limits_{K} \,\mathop {\max }\limits_{M} V\left( {M,K} \right) = F_{{y \sim p\,data^{\left( y \right)} }} \left[ {\log M\left( {y|x} \right)} \right] + F_{{z \sim p_{z}^{\left( z \right)} }} \left[ {\log \left( {1 - M\left( {K\left( {z|x} \right)} \right)} \right)} \right]$$

Condition y differs for tasks and datasets, while condition x should be the same for multiple samples in the same category for a given task and dataset.

CycleGAN performs superior to the earlier techniques for the image translation job, which requires pair datasets. After applying a GAN to turn image A into an image, we notice distinct styles in images A and BB. The visual translation is reversible. To translate the image, we may also apply a different GAN. CycleGAN uses a cycle consistency loss function to guarantee that the procedure is reversible in both directions:

3 $${N}_{cyc}\left({K}_{BtoA},{K}_{AtoB},B,A\right)={F}_{b\sim B}\left[||{K}_{AtoB}\left({K}_{BtoA}\left(b\right)\right)-b|{|}_{1}\right]+{F}_{a\sim A}\left[||{K}_{BtoA}\left({K}_{AtoB}\left(a\right)\right)-a|{|}_{1}\right]$$

where ${K}_{BtoA}$ and ${K}_{AtoB}$ stand for two different generators, b, and a, for images that fall under style B and A, respectively, and $||.|{|}_{1}$ for the L1-norm. CycleGAN's loss function is definite as surveys:

4 $$N\left({K}_{BtoA},{K}_{AtoB},{M}_{B},{M}_{A}\right)={N}_{K}\left({K}_{BtoA},{M}_{A},B,A\right)+{N}_{K}\left({K}_{AtoB},{M}_{B},A,B\right) +{N}_{cyc}\left({K}_{BtoA},{K}_{AtoB},B,A\right)$$

where ${N}_{K}$ stands for the GAN loss, ${N}_{cyc}$ for the cycle consistency loss and the discriminators corresponding to the generators ${K}_{BtoA}$ and ${K}_{AtoB}$, correspondingly.

Other techniques for translating images, called image-to-image translation, based on GAN, include DiscoGAN, DualGAN, and pix2pix.

#### The organization of the forest extraction generative adversarial network

We use CGAN to extract trees from aerial images because of its remarkable performance in image translation jobs. Using this method, we can create labeled images from aerial image. We can quickly change the visual properties of images by utilizing the capabilities of CycleGAN and other CGAN models. For example, we can convert items like a horse into a zebra, change the time of day from day to night, convert a summer setting into a winter scene, and more.

Here, the objective is to determine if a specific pixel is part of a forest or the background. This work aims to use the appropriate method to convert the aerial image into a binary representation of the forest and its surroundings.

Our model extracts the forest from aerial images using DCGAN and CGAN. We use a DCGAN structure under particular circumstances, exclusively using aerial images devoid of random noise. We utilize FCN rather than deconvolution layers because our input and output images are the same size. The DCGAN discriminator shares the same structure as this one. The blocks' numbers correspond to the feature maps for each tier. We add low-level features to the up-sampled feature maps in our FCN model, which has the FCN-4s structure. Comparing our FCN network to the conventional FCN-4s model reveals that we have a different number of layers and feature maps and that we have eliminated the pooling layers for downsampling.

The FCN part's structure offers a variety of options. Due to Unet's more complex architecture and effective performance in forest extraction, we apply it here. We do not have a pooling layer in our system. Instead, the Unet architecture uses eight convolutional or deconvolutional layers with asymmetric down- and up-sampling layers grouped in layers.

#### Loss function

There is no need for two GANs or the inclusion of cycle consistency, as seen in CycleGAN, because our forest dataset already contains corresponding aerial and binary images. Additionally, there is no need for image translation for the forest extraction operation. The following definition applies to our loss function:

5 $$N = \mathop {\arg \min }\limits_{K} \,\mathop {\max }\limits_{M} \alpha N_{cGAN} \left( {K,M} \right) + \beta N_{contenet} \left( K \right)$$

where and are hyper-parameters that balance the two distinct losses, and ${N}_{cGAN}$ stands for the CGAN loss, ${N}_{contenet}$ for the content loss.

Due to its simple nature, we initially selected an L1-norm loss function. The ${N}_{contenet}\left(K\right)={N}_{1}\left(K\right)={F}_{y\sim Y}||K\left(y\right)-x|{|}_{1}$ the function makes use of several elements, including 1. denotes the N1 distance, K(y) represents the binary image created from the aerial image used as input, X represents the ground truth, and Y defines the aerial image that was included in the training batch ${F}_{y\sim Y}||K\left(y\right)-x|{|}_{1}$. The following is a way to put this.

6 $${F}_{y\sim Y}||K\left(y\right)-x|{|}_{1}=\frac{1}{c}{\sum }_{i=1}^{c}{\sum }_{j=1}^{C\times O}|K{\left({y}^{i}\right)}_{j}-{x}_{j}^{i}|$$

The index of the samples in the current batch is represented by "i," the different example is symbolized by "k," the index of pixels within each image is signified by "j," and the image size is referred to as $C\times O$. For the training batch size. The following is how to express our model's loss function when using the N1-norm:

7 $$N = \arg \mathop K\limits^{\min } \mathop M\limits^{\max } \frac{1}{c}\sum\limits_{i = 1,k \ne i}^{d} {\sum\limits_{j = 1}^{D \times O} {\left( {\alpha \left( {\log M\left( {x^{k} |y^{k} } \right) + \log \left( {1 - M\left( {K\left( {y^{i} } \right)} \right)} \right)} \right) + \beta \left| {K\left( {y^{i} } \right)_{j} - x_{j}^{i} } \right|} \right)} }$$

Another option is the N2-norm loss function, which may be expressed as Eq. (8) for ${N}_{contenet}\left(K\right)={N}_{2}\left(K\right)={F}_{y\sim Y}{\Vert K\left(y\right)-x\Vert }_{2}$ and ${F}_{y\sim Y}{\Vert K\left(y\right)-x\Vert }_{2}$. Equation (9) can be used to write the loss function for our model with N2 loss.

8 $${F}_{y\sim Y}{\Vert K\left(y\right)-x\Vert }_{2}=\frac{1}{d}{\sum }_{i=1}^{d}\sqrt{{\sum }_{j=1}^{D\times O}{\left(K{\left({y}^{i}\right)}_{j}-{x}_{j}^{i}\right)}^{2}}$$

9 $$N = \arg \mathop K\limits^{\min } \mathop M\limits^{\max } \frac{\alpha }{c}\sum\limits_{i = 1,k \ne i}^{d} {\sum\limits_{j = 1}^{D \times O} {\left( {\log M\left( {x^{k} |y^{k} } \right) + \log \left( {1 - M\left( {K\left( {y^{i} } \right)} \right)} \right)} \right) + \frac{\beta }{c}\sum\limits_{i = 1}^{c} {\sqrt {\sum\limits_{j = 1}^{C \times O} {\left( {K\left( {y^{i} } \right)_{j} - x_{j}^{i} } \right)^{2} } } } } }$$

Our model's output was assessed using both N1 and N2 norms. We ultimately chose the N2 loss as our element-wise loss function, even though both the N1 and N2 loss functions generated satisfactory results. The experimental part will include a thorough comparison.

Lastly, we employ Eq. (9) as the loss function for our suggested technique for extracting forest from aerial images.

### YOLOv5 model

**Figure 5:**
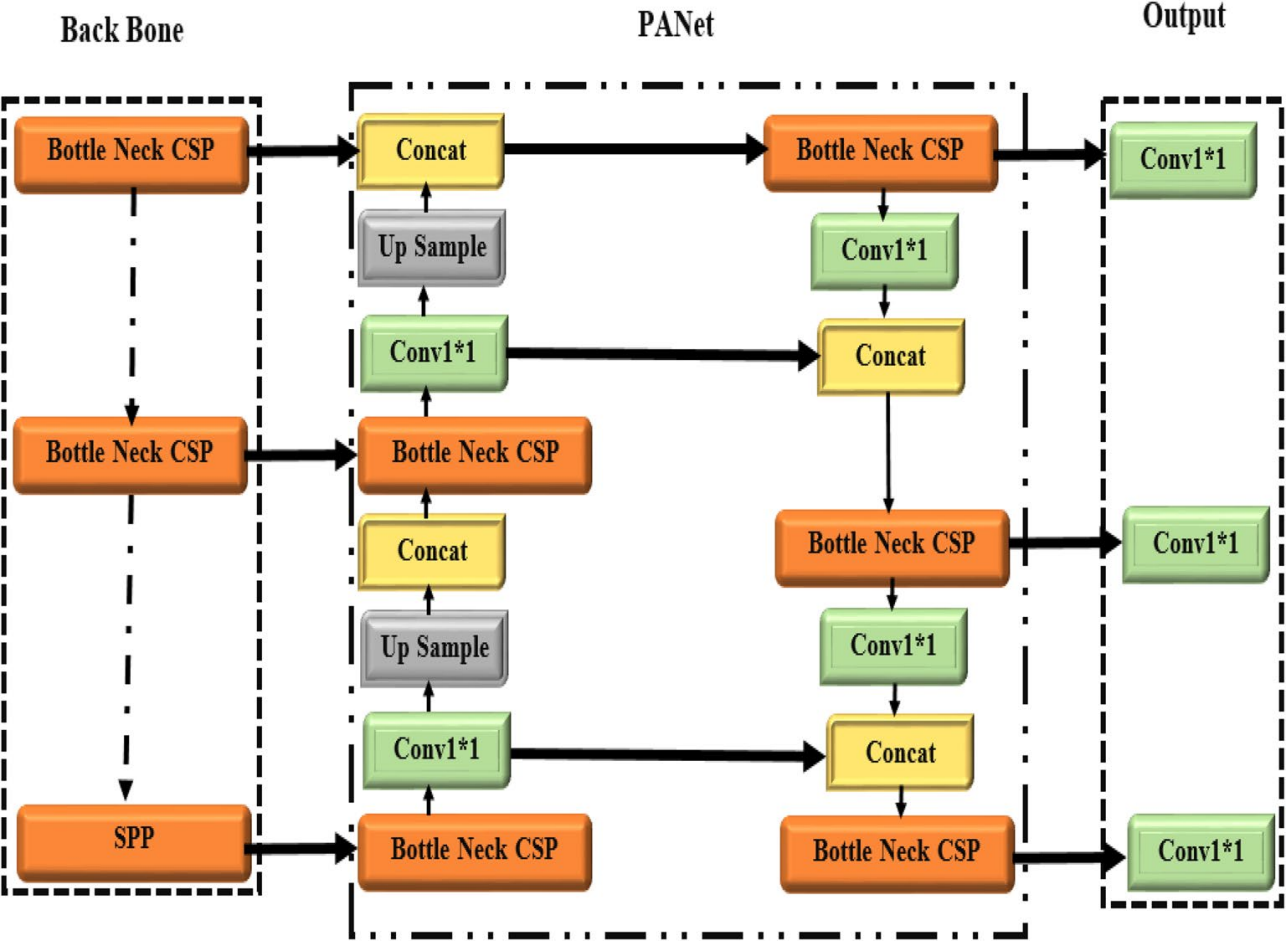
Architecture of YOLOv5 model.

The three object identification algorithms most often used in research are R-CNN, Faster RCNN, and YOLO. The YOLO series is preferred to the older iterations because of its quicker processing time and enhanced ability to perceive minute details. The YOLOv5 model was used in this investigation in numerous iterations for both training and testing. A wide range of object recognition models previously trained on the MS COCO dataset are included in the 2020 version of YOLOv5. YOLOv5 comes in five different flavours, each responding to a different set of requirements. Starting with the little YOLOv5 micro, which is designed for mobile and embedded devices, the options go to the large YOLOv5x. Figure 5 depicts the architectural components of YOLOv5, which include its head, neck, backbone, and other essential sections.

**Figure 6:**
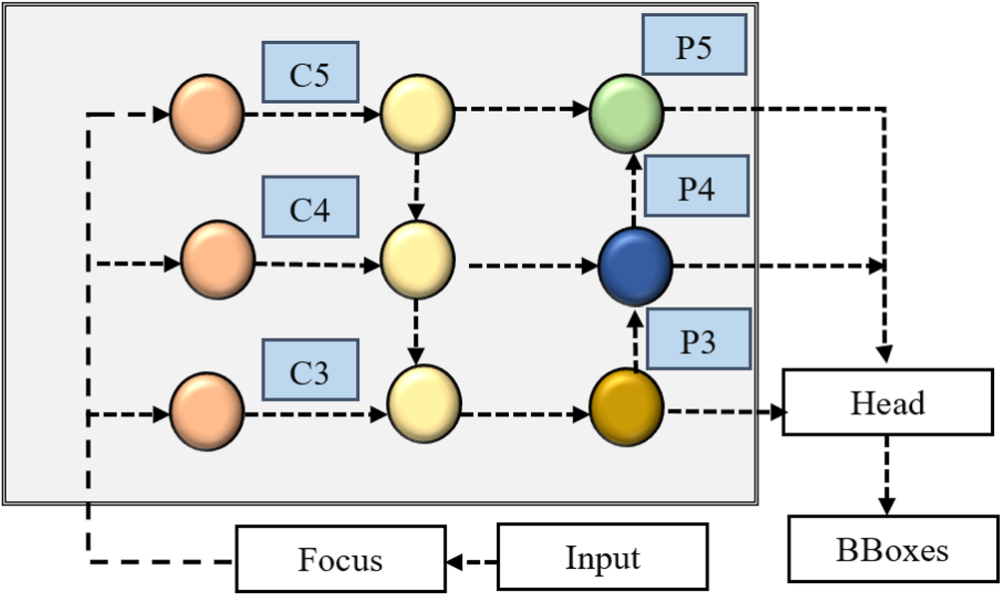
Representation of feature fusion of YOLOv5 model.

YOLOv5 contains important architectural modifications, including the focus structure, Cross Stage Partial Networks (CSP), and other components to improve performance. The backbone, which is made up of the focal structure and the CSP, is critical in processing the input data. The focal structure in the backbone manages input data downsampling while keeping the original data intact. The CSP Network, on the other hand, intelligently accumulates essential data, contributing to better learning capacities while without consuming excessive memory. The model then analyses the relevant features through the neck section, combining the Path Aggregation Network (PAN) and Feature Pyramid Networks (FPN). PAN enables the integration of features from many levels, resulting in more accurate forecasts. Meanwhile, FPN uses prediction fusion and top-to-bottom communication to upsample high-level feature data. The underlying pyramid architecture of the PAN allows critical key location information to cascade from the top to bottom layers. This enables the model to detect objects of varied scales and sizes, resulting in improved performance, particularly with recent data. Bounding boxes, class probabilities, and object scores are among the final output vectors generated by the output layer's head using anchor boxes applied to the features. The addition of the focus and CSP layers is the most significant improvement in YOLOv5. By lowering the number of layers, parameters, FLOPS, and CUDA memory usage, the focusing layer minimizes computational complexity, resulting in faster forward and backward passes. The CSP layer in the backbone of the YOLOv5 architecture is in charge of extracting specific data and conducting more intricate actions. Furthermore, the meshing concepts of the original YOLO algorithm are retained in YOLOv5. Overall, YOLOv5 has a streamlined architecture is depict in Fig. 6 with enhanced efficiency and performance, making it a big step forward from its predecessors.

The network takes an RGB image as an input and produces a three-scale output. The Process of bounding box regression of YOLOv5 can be explained in detail by Eq. (10)

10 $${a}_{x}=2\sigma \left({m}_{x}\right)-\frac{1}{2}+{r}_{x}$$

11 $${a}_{y}=2\sigma \left({m}_{y}\right)-\frac{1}{2}+{r}_{y}$$

12 $${a}_{h}={q}_{h}{\left(2\sigma \left({m}_{h}\right)\right)}^{2}$$

13 $${a}_{w}={q}_{w}{\left(2\sigma \left({m}_{w}\right)\right)}^{2}$$

For the upper-left corner of the feature map, use the coordinates (0, 0) in the aforementioned calculation. The values $${r}_{\begin{array}{c}x\\ \end{array}}$$ and $${r}_{\begin{array}{c}y\\ \end{array}}$$ denote the distance from the top left corner of the grid to the centre of the label's bounding box. The coordinates ${a}_{x}$ and ${a}_{y}$ designate the label's centre, while ${a}_{h}$ and ${a}_{w}$ define its width and height. The parameters ph and pw are based on the dimensions of the preceding bounding box. The parameters ${m}_{x}$, ${m}_{y}$, ${m}_{h}$, and ${m}_{w}$ define the limits of the bounding box.

## Result and discussion

### Experimental setup

The testing dataset was a combination of the training and validation datasets. This was accomplished using a desktop computer outfitted with 64 GB of RAM, an Intel Core i7-10700 CPU, and an NVIDIA GeForce RTX 2070 graphics card. We used transfer learning and data augmentation approaches to evaluate the generalization of our model because the network wasn't present during the evaluation phase. Each of the 25 epochs that made up the training procedure had 100 training steps and 25 validation steps. The model was trained using a 0.001 learning rate. We evaluate Mask-RCNN^27,28^, Faster-RCNN^29,30^, YOLO V3^31,32^, and YOLO V5^33,34^ in comparison to our suggested system.

### Ablation experiment

In the ablation study conducted on MGAN-YOLOV5 for Automated Forest Health Diagnosis from Aerial Imagery, several experiments were carried out to assess the individual impact of different components on the model's performance. The baseline MGAN-YOLOV5, achieving an mAP (mean Average Precision) of 87%, served as the starting point. The experiments focused on isolating and removing specific elements within the architecture. These included the removal of YOLO V5techniques, such as random scaling and flipping, resulting in a decrease in performance to 82% mAP. Additionally, the absence of the YOLO V3 led to a noticeable reduction in accuracy, with a mAP of 78%. A slightly smaller impact was obtained with the removal of the MGAN, yielding a mAP of 83%. The ablation study results can provide light on the significance of the TSA is mAP of 84% in the context of automated forest health diagnosis from aerial imagery Using MGAN with YOLOv5. Removal of the Faster-RCNN resulted in a slightly lower impact, achieving a mAP of 84%. Finally, the absence of Mask-RCNN training data resulted in a mAP of 85%. These experiments demonstrated the significance of individual components in the MGAN-YOLOV5 model for forest health diagnosis from aerial imagery, emphasizing the crucial role each element plays in overall model performance. The results of the ablation experiments on the classification task of the backbone are shown in Table 2.

**Table 2:** Results of the ablation experiment.

Models	mPA (%)
Mask-RCNN	85
Faster-RCNN	84
YOLO V3	78
YOLO V5	82
TSA	84
MGAN	83
MGAN-YOLOV5	87

The use of YOLOv5 improves real-time object detection capabilities, allowing for quick and precise identification of several forest health indices. The redesigned GANs are critical in producing synthetic data, which increases the diversity of the training dataset and improves the model's capacity to generalize to a wider range of situations. This synthesis capacity not only helps to address data shortage difficulties, but it also contributes to the model's robustness against unexpected fluctuations. Furthermore, the TSA enhances the model's hyperparameters and training process, allowing for more efficient convergence and better overall performance. This optimization technique ensures that the model is properly adjusted for the specific purpose of determining forest health, resulting in improved accuracy and reliability. The combination of these components produces a comprehensive and customizable framework that outperforms previous methods in the automated assessment of forest health using aerial images. TSA systematically explores the search space, adjusting hyperparameters and training methods to direct the model to near-optimal solutions. This comprehensive investigation ensures improved convergence, which raises the precision of disease diagnosis while decreasing false positives.

### Performance metrics

The proportion of accurately diagnosed samples classified as positive is known as precision (P).

14 $$P=\frac{TP}{TP+FP}$$

Recall (R) measures how many positive samples were correctly classified out of all the positive samples.

15 $$\mathit{Re}call=\frac{TP}{TP+FN}$$

Accuracy: this metric measures the number of cases that are correctly categorized. Performance is best when there are equivalent examples in each class or when the types are balanced. It is calculated using Eq. (16).

16 $$Accuracy=\frac{TP+TN}{TP+FN+TN+FP}*100$$

Mean average precision (mAP): the mean average precision (mAP) is then derived by calculating the average of the AP values. mAP is considered a crucial metric for assessing the overall precision of the target detection model, making it a reliable indicator of its performance. It is calculated using Eq. (18)

17 $$AP = \int {P(R)\,dR}$$

18 $$mAP = \frac{1}{c}\,\sum\limits_{j}^{c} {APj}$$

#### Precision analysis

**Figure 7:**
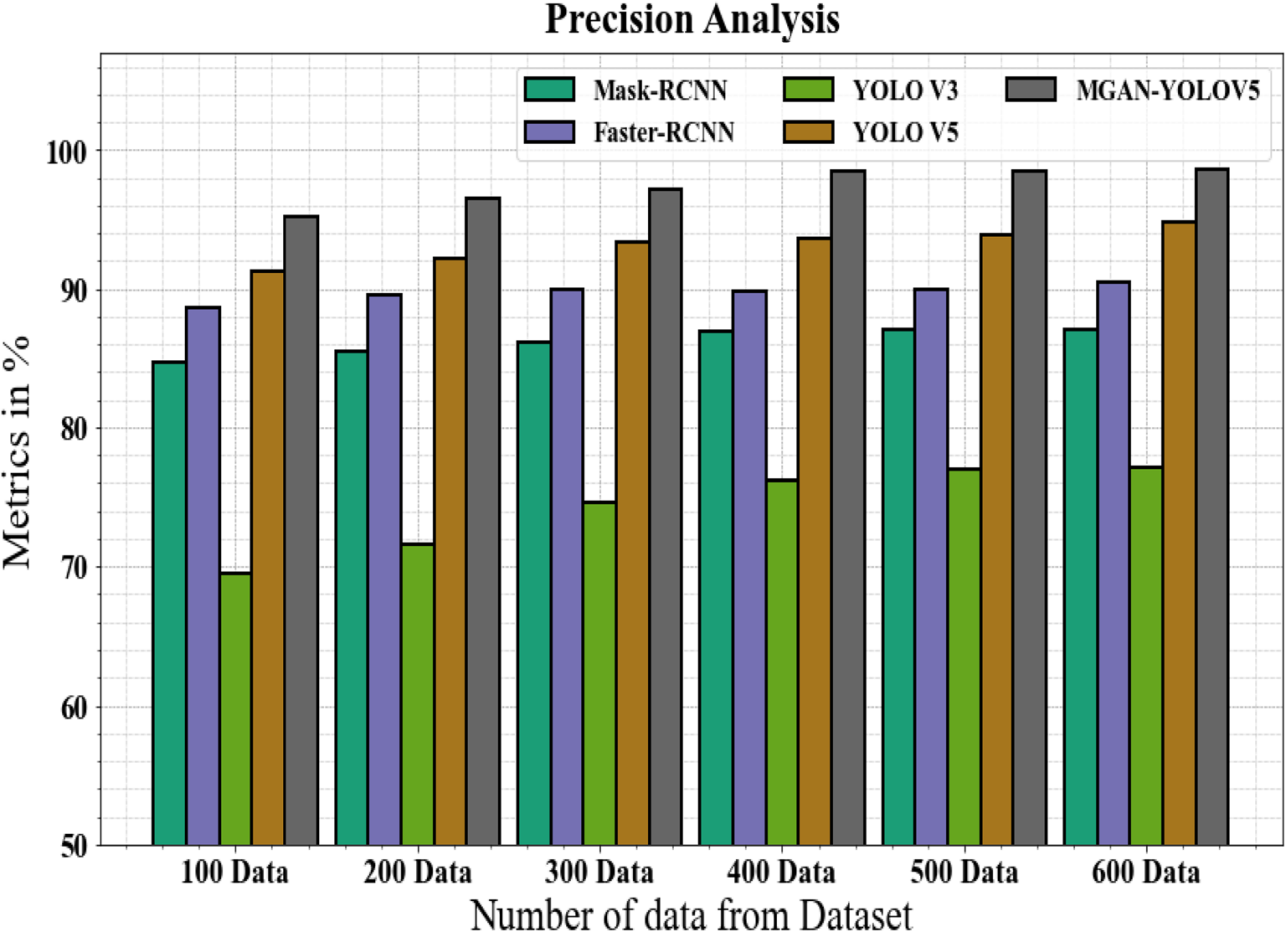
Precision analysis for MGAN-YOLOV5 method with existing systems.

In Fig. 7 and Table 3, the MGAN-YOLOV5 methodology's precision is contrasted with other approaches. The graph shows how the deep learning method boosts precision with efficiency. For instance, the Mask-RCNN, Faster-RCNN, YOLO V3, and YOLO V5 models' respective precision values for 100 data are 84.67%, 88.66%, 69.56%, and 91.23%, as opposed to the MGAN-YOLOV5 model's precision with 95.19%. The MGAN-YOLOV5 model, however, has shown to work best with various data sizes. Like this, the precision for MGAN-YOLOV5 under 600 data is 98.66%, while those for Mask-RCNN, Faster-RCNN, YOLO V3, and YOLO V5 are 87.12%, 90.45%, 77.12%, and 94.77%, correspondingly.

**Table 3:** Precision analysis for MGAN-YOLOV5 method with existing systems.

Number of data from dataset	Mask-RCNN	Faster-RCNN	YOLO V3	YOLO V5	MGAN-YOLOV5
100	84.67	88.66	69.56	91.23	95.19
200	85.55	89.56	71.56	92.19	96.55
300	86.12	89.91	74.67	93.33	97.12
400	86.98	89.76	76.18	93.56	98.45
500	87.11	89.99	76.99	93.87	98.51
600	87.12	90.45	77.12	94.77	98.66

#### Recall analysis

**Figure 8:**
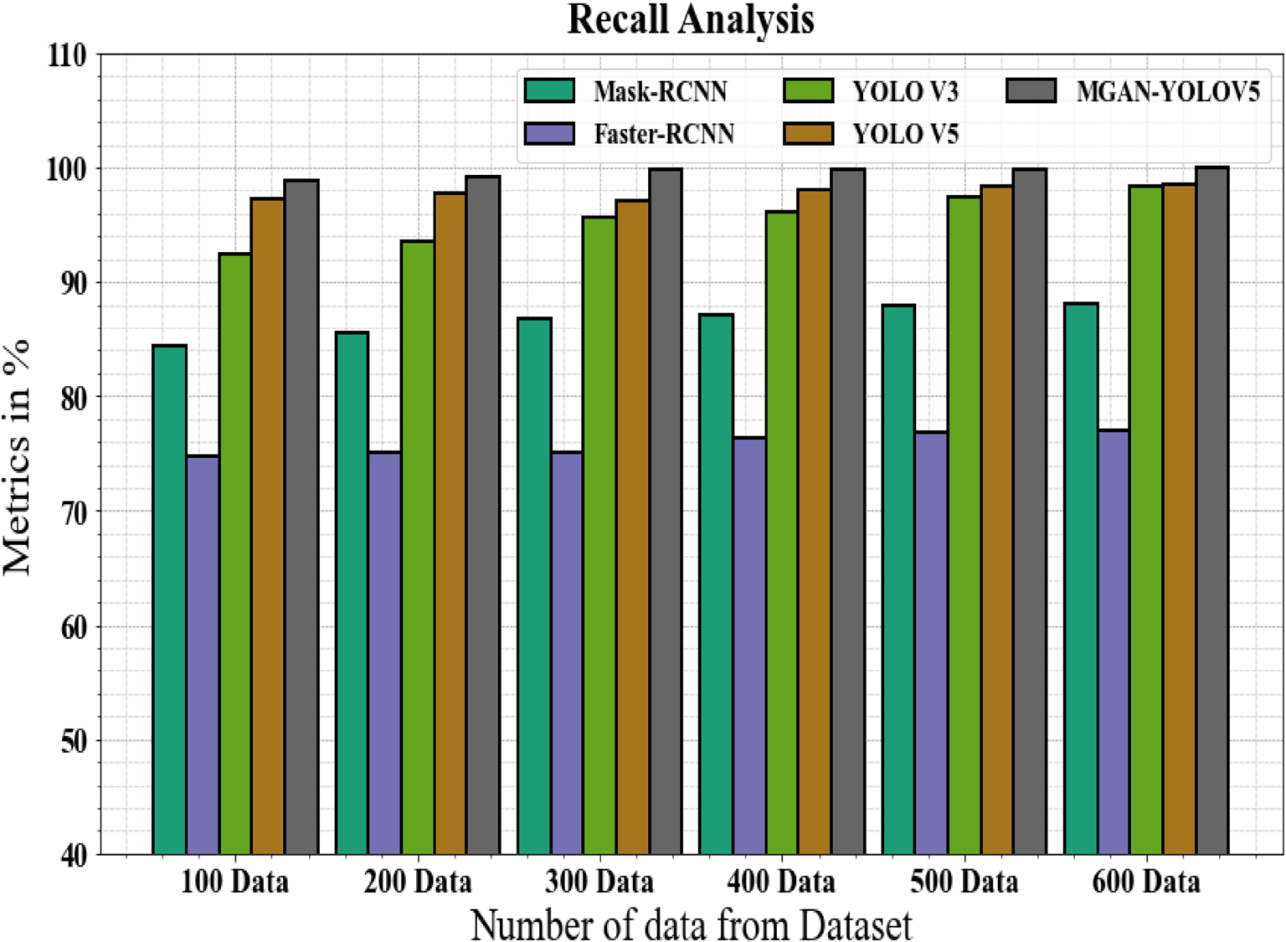
Recall analysis for MGAN-YOLOV5 method with existing systems.

The recall of the MGAN-YOLOV5 methodology is compared to other methods in Fig. 8 and Table 4. The graph demonstrates the higher recall efficiency for the deep learning approach. For example, the Mask-RCNN, Faster-RCNN, YOLO V3, and YOLO V5 models' respective recall values for 100 data are 84.34%, 74.78%,92.45%, and 97.34%, respectively, as opposed to the MGAN-YOLOV5 model's recall value of 98.89%. The MGAN-YOLOV5 model, however, has shown to work best with various data sizes. Like this, the recall value for MGAN-YOLOV5 under 600 data is 99.99%, while those for Mask-RCNN, Faster-RCNN, YOLO V3, and YOLO V5 are 88.12%, 76.91%,98.43%, and 98.57%, correspondingly.

**Table 4:** Recall analysis for MGAN-YOLOV5 method with existing systems.

Number of data from dataset	Mask-RCNN	Faster-RCNN	YOLO V3	YOLO V5	MGAN-YOLOV5
100	84.34	74.78	92.45	97.34	98.89
200	85.56	74.98	93.56	97.78	99.19
300	86.87	75.12	95.59	97.11	99.78
400	87.11	76.34	96.12	98.12	99.89
500	87.98	76.89	97.43	98.45	99.91
600	88.12	76.91	98.43	98.57	99.99

#### F1-score analysis

**Figure 9:**
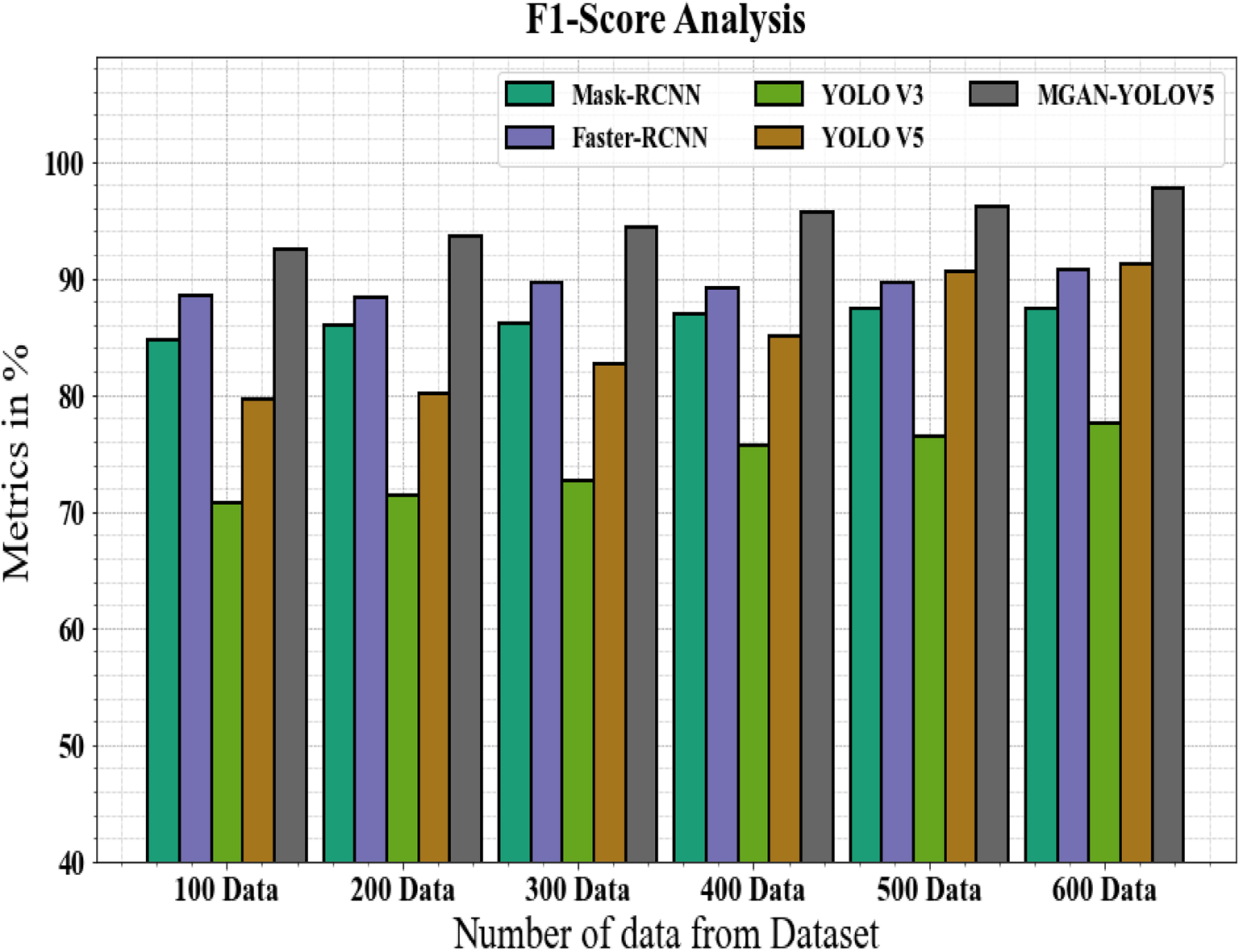
F1-Score analysis for MGAN-YOLOV5 method with existing systems.

The f1-score of the MGAN-YOLOV5 methodology is compared to that of other techniques in Fig. 9 and Table 5. The graph establishes how the deep learning approach increases the f1-score efficiency. For instance, the Mask-RCNN, Faster-RCNN, YOLO V3, and YOLO V5 models' respective f1-score values for 100 data are 84.67%, 88.56%,70.67%, and 79.66%, respectively, as opposed to the MGAN-YOLOV5 model's f1-score value of 92.56%. However, the MGAN-YOLOV5 model has shown to perform best with numerous data. Like this, under 600 data, the MGAN-YOLOV5 has the f1-score value of 97.77%, while the corresponding f1-score values for Mask-RCNN, Faster-RCNN, YOLO V3, and YOLO V5 are 87.45%, 90.67%, 77.56%, and 91.23%.

**Table 5:** F1-score analysis for MGAN-YOLOV5 method with existing systems.

Number of data from dataset	Mask-RCNN	Faster-RCNN	YOLO V3	YOLO V5	MGAN-YOLOV5
100	84.67	88.56	70.67	79.66	92.56
200	85.98	88.34	71.34	80.12	93.67
300	86.12	89.67	72.67	82.67	94.44
400	86.98	89.12	75.67	84.98	95.67
500	87.45	89.56	76.45	90.56	96.11
600	87.45	90.67	77.56	91.23	97.77

#### Accuracy analysis

**Figure 10:**
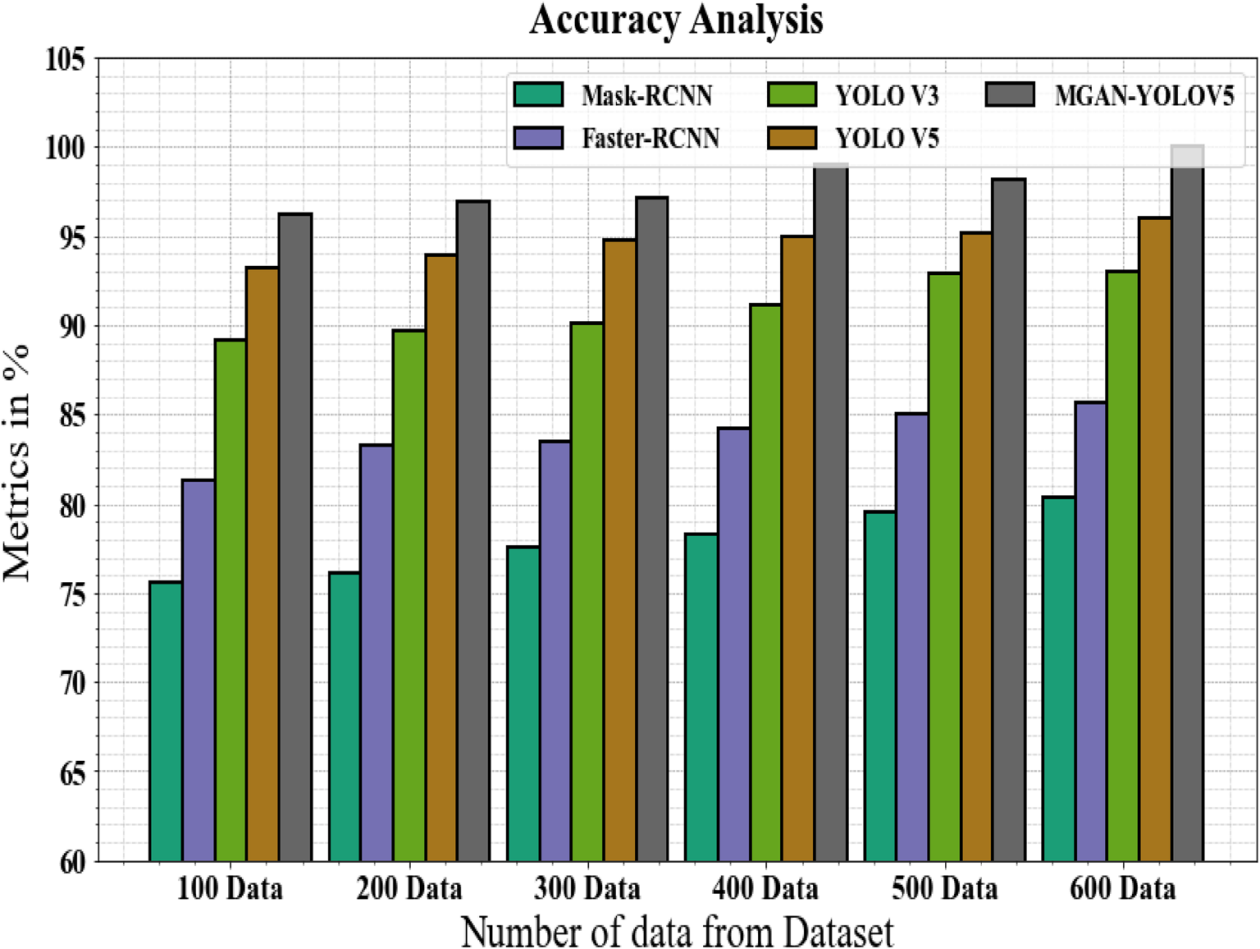
Accuracy analysis for MGAN-YOLOV5 method with existing systems.

Figure 10 and Table 6 associate the MGAN-YOLOV5 methodology's accuracy with other methods. The graph demonstrates the increased accuracy of the deep learning approach. For instance, the Mask-RCNN, Faster-RCNN, YOLO V3, and YOLO V5 models' respective accuracy values for 100 data are 75.56%, 81.34%, 89.13%, and 93.19%, respectively, as opposed to the MGAN-YOLOV5 model's accuracy of 96.17%. However, the MGAN-YOLOV5 model has shown to perform best with numerous data. Like this, under 600 data, the MGAN-YOLOV5 has an accuracy of 99.99%, while the corresponding accuracy values for Mask-RCNN, Faster-RCNN, YOLO V3, and YOLO V5 are 80.34%, 85.67%, 92.99%, and 95.98%.

**Table 6:** Accuracy analysis for MGAN-YOLOV5 method with existing systems.

Number of data from dataset	Mask-RCNN	Faster-RCNN	YOLO V3	YOLO V5	MGAN-YOLOV5
100	75.56	81.34	89.13	93.19	96.17
200	76.12	83.29	89.66	93.89	96.89
300	77.56	83.44	90.14	94.78	97.16
400	78.34	84.19	91.11	94.99	98.98
500	79.55	84.99	92.87	95.12	98.19
600	80.34	85.67	92.99	95.98	99.99

#### Response time analysis

**Figure 11:**
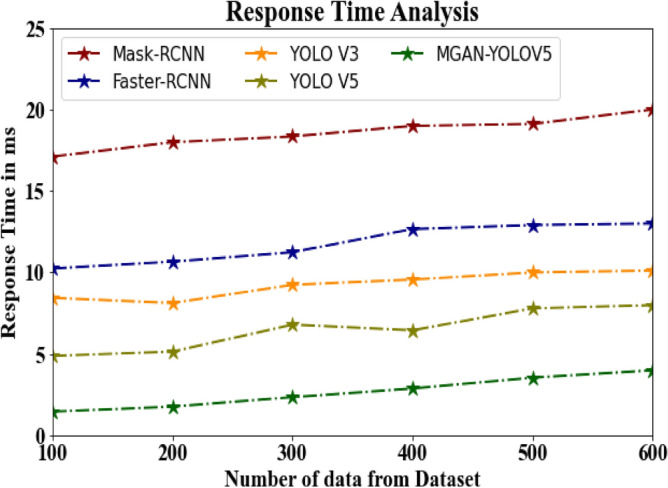
Response time analysis for MGAN-YOLOV5 method with existing systems.

The database response time of the proposed MGAN-YOLOV5 strategy is compared to known approaches in Table 7 and Fig. 11. The statistics show that the proposed MGAN-YOLOV5 strategy outperformed all other techniques combined. The suggested MGAN-YOLOV5 approach has taken only 1.456 ms to respond, whereas other current methods such as Mask-RCNN, Faster-RCNN, YOLO V3, and YOLO V5 took 17.113 ms, 10.234 ms, 8.432 ms, and 4.876 ms, respectively as their response time. Similarly, the suggested MGAN-YOLOV5 approach takes 3.987 ms to respond to 600 data, while the existing techniques like Mask-RCNN, Faster-RCNN, YOLO V3, and YOLO V5 have taken 19.993 ms, 12.994 ms, 10.111 ms, and 7.991 ms respectively as their response time.

**Table 7:** Response time analysis for MGAN-YOLOV5 method with existing systems.

Number of data from dataset	Mask-RCNN	Faster-RCNN	YOLO V3	YOLO V5	MGAN-YOLOV5
100	17.113	10.234	8.432	4.876	1.456
200	17.987	10.654	8.119	5.145	1.765
300	18.345	11.234	9.234	6.789	2.345
400	18.897	12.654	9.556	6.443	2.876
500	19.112	12.897	9.998	7.789	3.543
600	19.993	12.994	10.111	7.991	3.987

#### Computational time analysis

**Figure 12:**
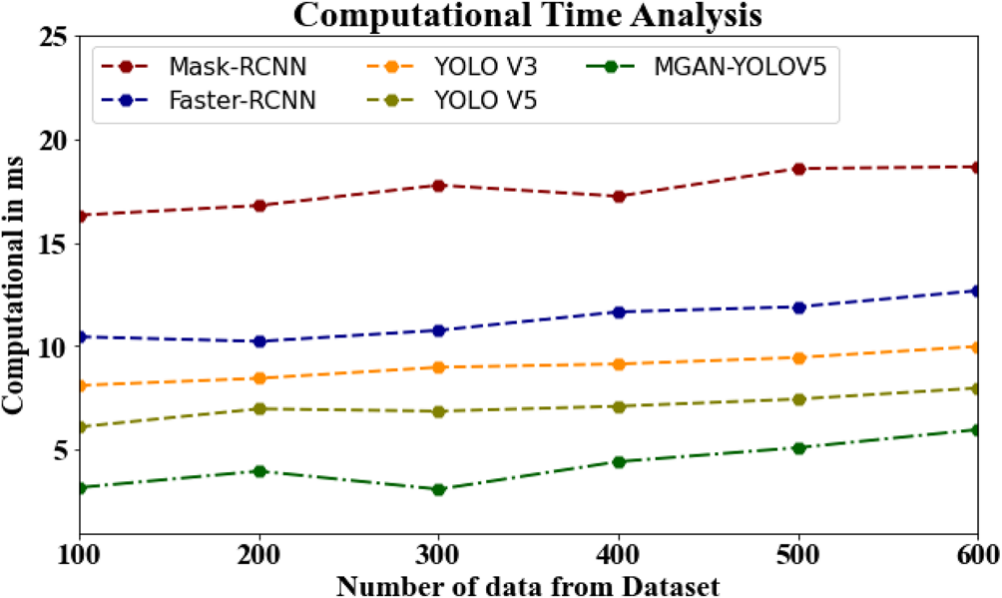
Computational time analysis for MGAN-YOLOV5 method with existing systems.

The database computational time of the proposed MGAN-YOLOV5 strategy is compared to known approaches in Table 8 and Fig. 12. The statistics show that the proposed MGAN-YOLOV5 strategy outperformed all other techniques combined with less computational time. The suggested MGAN-YOLOV5 approach has taken only 3.198 ms to compute 100 data, whereas other current methods such as Mask-RCNN, Faster-RCNN, YOLO V3, and YOLO V5 have taken 16.324 ms, 10.456 ms, 8.115 ms, and 6.119 ms, respectively as their computational time. Similarly, the suggested MGAN-YOLOV5 approach takes only 5.987 ms to compute 600 data, while the existing techniques like Mask-RCNN, Faster-RCNN, YOLO V3, and YOLO V5 have taken 18.654 ms, 12.675 ms, 9.991 ms, and 7.897 ms respectively as their computational time.

**Table 8:** Computational time analysis for MGAN-YOLOV5 method with existing systems.

Number of data from dataset	Mask-RCNN	Faster-RCNN	YOLO V3	YOLO V5	MGAN-YOLOV5
100	16.324	10.456	8.115	6.119	3.198
200	16.786	10.234	8.456	6.987	3.987
300	17.765	10.765	8.987	6.876	3.115
400	17.234	11.654	9.145	7.113	4.445
500	18.567	11.897	9.456	7.456	5.116
600	18.654	12.675	9.991	7.897	5.987

## Conclusion

Finally, we present a novel framework for automatically identifying dead trees in aerial images. This approach uses a transfer learning strategy in conjunction with a re-engineered MGAN-YOLOv5 technology. We use this approach to compare eight painstakingly developed models using our collection of aerial imaging datasets. A phenomenal precision score of 98% is attained by the model that stands out above the rest. Additionally, we can design mask visualizations that highlight dead trees in an image. With the help of this development, we can now quantitatively determine how many dead trees are present in a specific area. Using aerial image-based forest analysis, we can proactively assess forest health, identify potential risk factors, and recommend practical solutions to lessen climate change-related disasters, like the recent forest fires in California and Australia. Aerial images is used in this paper as a novel tool for measuring forest health. The TSA is also used to improve the finding and categorizing of hazardous forest regions. The MGANs architecture is intended to deal with complicated aerial imagery, such as fluctuations in illumination, canopy coverage, and topography. The suggested model adds synthetic data to the restricted labeled dataset, addressing the common data scarcity issue in forest health detection tasks. This enhancement improves the model's capacity to generalize to previously unseen variables, boosting the overall precision and resilience of the forest health assessment. Furthermore, YOLOv5 integration allows real-time object recognition, allowing the model to recognize and localize multiple tree species and potential health issues with extraordinary speed and accuracy. The reduced architecture of YOLOv5 enables its deployment on devices with limited resources, enabling real-time forest monitoring on the spot. The TSA is used to improve the detection of unhealthy forest regions. The TSA technique effectively examines the search space, ensuring the model converges to a near-optimal solution, improving disease diagnosis precision and lowering false positives. Using a huge dataset of aerial images of various forest environments, we tested our MGAN-YOLOv5 technique. We plan to analyze multiple remote sensing data, including images and MGAN-YOLOv5 data, to evaluate the decay rates of standing dead trees.

## Data Availability

The datasets used during the current study are available from the corresponding author on reasonable request.
